# Effects of maternal education on diet, anemia, and iron deficiency in Korean school-aged children

**DOI:** 10.1186/1471-2458-11-870

**Published:** 2011-11-16

**Authors:** Hyeon-Jeong Choi, Hye-Ja Lee, Han Byul Jang, Ju Yeon Park, Jae-Heon Kang, Kyung-Hee Park, Jihyun Song

**Affiliations:** 1 Division of Metabolic Diseases, Korea National Institute of Health, Cheongwon-gun, Chungcheongbuk-do 363-951, South Korea; 2 Department of Family Medicine, Obesity Research Institute, Seoul-Paik Hospital, Inje University, Seoul 100-032, South Korea; 3 Department of Family Medicine, Hallym University Sacred Heart Hospital, Hallym University, Anyang, Gyeonggi-do 431-796, South Korea

## Abstract

**Background:**

We investigated the relationship among socioeconomic status factors, the risk of anemia, and iron deficiency among school-aged children in Korea.

**Methods:**

The sample consisted of fourth-grade students aged 10 y recruited from nine elementary schools in Korean urban areas in 2008 (*n *= 717). Anthropometric and blood biochemistry data were obtained for this cross-sectional observational study. Anemia was defined as hemoglobin levels lower than 11.5 g/dl. Iron deficiency was defined as serum iron levels lower than 40 ug/dl. We also obtained data on parental education from questionnaires and on children's diets from 3-day food diaries. Parental education was categorized as low or high, with the latter representing an educational level beyond high school.

**Results:**

Children with more educated mothers were less likely to develop anemia (*P *= 0.0324) and iron deficiency (*P *= 0.0577) than were those with less educated mothers. This group consumed more protein (*P *= 0.0004) and iron (*P *= 0.0012) from animal sources than did the children of less educated mothers, as reflected by their greater consumption of meat, poultry, and derivatives (*P *< 0.0001). Logistic regression analysis revealed a significant inverse relationship between maternal education and the prevalence of anemia (odds ratio: 0.52; 95% confidence interval: 0.32, 0.85).

**Conclusions:**

As a contributor to socioeconomic status, maternal education is important in reducing the risk of anemia and iron deficiency and in increasing children's consumption of animal food sources.

## Background

According to the World Health Organization, anemia is a public health problem in many developing countries, including Africa and Asia [1]. Most studies on anemia have been performed in developing countries and have focused on certain groups, such as children younger than 5 years of age. The prevalence of anemia in school-aged children has received less attention compared with that in preschoolers and women of child-bearing age [2,3]. However, the prevalence of anemia in school-aged children has been estimated to be as high as 9% in some industrialized, developed countries [4].

Anemia is an indicator of both poor nutrition and health [5], and it affects cognitive functioning, motor performance, and educational achievement [6,7]. Iron deficiency is caused by the insufficient intake and absorption of iron and is the most common cause of anemia. Iron absorption is influenced by the storage of iron in the body, the form of dietary iron, and other dietary factors [8]. For example, vitamin C is generally believed to facilitate iron utilization, although this has not been proven [9,10]. Many studies have reported a negative correlation between the consumption of cow's milk and iron status during childhood [11,12] and an inhibitory effect of dietary fiber on non-heme iron absorption [8,13].

In addition to dietary factors, household characteristics such as hygiene, parental education, household income, and number of children in the family are also important determinants of nutritional knowledge and diet [14,15]. A strong relationship may exist between a child's health and the educational level of his or her parents [14]. Higher maternal education has been shown to lead to increased knowledge about health and nutrition and to an increase in the quality of the diets of children [15]. Maternal education may also affect healthy decision making and thus influence the probability of a child meeting certain nutrition-related requirements [16]. Research in developing countries has shown that children of formally educated [17] or literate [18] mothers had a reduced risk of stunting. In contrast, paternal education was a stronger determinant of child stunting than maternal education in Bangladesh [14] and the Philippines [19].

Because childhood anemia and iron deficiency have a significant impact on life-long health, it is important to identify determinants of this condition early in the child's life. If the iron requirements of a growing child are not fulfilled, learning ability, work efficiency, and immune functioning will decline prior to the emergence of other signs of anemia. If the iron deficiency persists, it can also result in height and weight disturbances, retardation of behavior and learning, and disturbances in physical or mental growth [20]. Early detection and subsequent preventive measures can help the child grow into a healthy adult.In the present study, we investigated the relationship between socioeconomic status factors, the diet of school-aged children, the risk of anemia, and iron deficiency in Korea.

## Methods

### Subjects

Fourth-grade students (*n *= 717) aged 10 y were recruited from nine elementary schools in the urban areas of Korea in 2008. This study was conducted as part of the Gwacheon Pediatric Cohort Study, which follows this student cohort from Gwacheon City in Kyunggi Province of Korea from their entry into elementary school at age 7 to their graduation at age 13. The objective of this pediatric cohort study was to identify early risk factors for obesity and associated metabolic diseases in urban Korean children. The sole inclusion criterion was enrollment in first grade, and no exclusion criteria were applied. This study was approved by the Institutional Review Board of Seoul-Paik Hospital of Inje University and by the Korean Center for Disease Control and Prevention (KCDC). Informed consent was obtained from the children's parents.

### Structured questionnaire

A questionnaire addressing family income, parental education, and food-consumption behaviors were sent to and completed at the homes of participants; children and their parents completed the questionnaires. According to the report on Economic Policy Directions for 2007, the Korean government (Ministry of Strategy and Finance) considers families to be middle class if their monthly income is 3,400,000 won. Thus, we categorized household income as low (< 3,000,000 won) or high (≥ 3,000,000 won).

### Anthropometric assessments

Height was measured by a trained technician using an automatic stadiometer (model DS 102, Jenix, Korea). Weight was measured by bioimpedence analysis using a body composition analyzer (TANITA, BC-418, Japan). Body mass index (BMI) was calculated by dividing weight in kilograms by height in meters squared. All those with a BMI ≥85th percentile were classified as overweight [21] using data from the 2007 KCDC growth chart [22].

### Biochemical data

Children's blood samples were collected from the antecubital vein and funneled into vacutainer tubes after a 12-h overnight fast. Hemoglobin (hb) concentrations, white blood cell counts (WBCs), and red blood cell counts (RBCs) were determined by flow-cytometry methods using the XE-2100D (Sysmex, Japan). Blood glucose was analyzed using the hexokinase method, and alanine aminotransferase (ALT) was measured using an enzymatic method with a Hitachi 7180 analyzer (HITACHI, Japan). Serum iron levels and unsaturated iron binding capacity (UIBC) were measured using the Ferrozine method (Roche Diagnostics, Germany) on a Cobas Integra 800 Analyzer (Roche, Switzerland). Total iron-binding capacity (TIBC) was calculated indirectly from the iron and the UIBC plus iron concentrations. We used an enzyme immunoassay (EIA) using the Human soluble transferring receptor (sTfR) immunoassay kit (R&D systems, USA) to determine sTfR. Serum ferritin was measured with a Chemiluminescence Immunoassay (CLIA) Analyzer (ACS 180, Bayer Diagnostics, USA). Transferrin saturation (TS (%)) was calculated by dividing the concentration of serum iron by the TIBC. Anemia was defined as a hemoglobin concentration of <11.5 g/dl [1], and iron deficiency was defined by a serum iron concentration of <40 μg/dl [23]. We had tried to define iron deficiency using sTfR and serum ferritin ratio. However, our sTfR and serum ferritin ratio could not give meaningful information based on the sensitivity and specificity estimated from receiver operating characteristics (ROC) curve (data not shown).

### Dietary assessment

The typical dietary intake of each child was estimated based on food records that were maintained by each child with help from his or her parents for 3 consecutive days (2 weekdays, 1 weekend day). Parents were also asked to maintain 3-day food records for their children. Recipes of school meals (lunches) were provided to the parents. Dietary questionnaires, which were certified by Seoul-Paik Hospital, Inje University, were confirmed as eliciting sufficient dietary information. Before starting the study, guideline of food record was provided for meaningful reply to children and their parents. The guideline was as follows: 1) Record your typical diet. 2) Include all beverages, meals, snacks, and tastes in total intake. 3) Be as specific as possible about the foods and drinks-seasonings, ingredients, preparation methods, and other details. 4) Record portion size using kitchen utensils, nutrition facts label, and other measurements-volume (1 cups, 1 tablespoons, 2 cm × 5 cm × 1 cm), weight (5 grams), size (small, medium, large), and count (two pieces of chocolate). Food intake was analyzed to nutrient intake using the CAN-pro 3.0 (Computer Aided Nutritional Analysis version 3.0 for professional) software program. The software was made for the purpose of easy and precise nutrition assessment and management by the Korean Nutrition Society. The software was included approximately 3,500 commonly consumed Korean foods and based on Korean Nutrient Database. To obtain the information for various nutrients in food, we entered food name, amount, and other details into the CAN-pro software and got the result of energy intake, macronutrients (carbohydrate, protein, fat), vitamin, mineral, and other nutrients. A set reference values for the dietary intake of Koreans was released in 2005 [24]; this consisted of four values: estimated average requirement (EAR), recommended intake (RI), adequate intake (AI), and tolerable upper intake level (UL). Low iron intake was defined as iron intake of less than the EAR (9 mg/day). Food groups were classified with reference to the food-group classification guidelines in the Korean Nutrient Database [24].

### Statistical methods

General characteristics were stratified based on factors related to socioeconomic status (SES) (maternal education, paternal education, and income level). Relationships among BMI, biochemical variables, hematological variables, and the factors comprising SES were assessed using a generalized linear model (GLM) adjusted for age and sex. Sex distributions and the frequencies of low iron intake and food-consumption behaviors by maternal education were analyzed with *chi*-square tests. The rates of anemia according to maternal education and the factors comprising SES were analyzed with Fisher's exact test. Nutrient intake and food-group consumption by maternal education were analyzed with the GLM and adjusted for age and sex. Finally, logistic regression was used to analyze children's risk of anemia and iron deficiency according to maternal education level. Statistical analyses were performed with the Statistical Analysis System software package version 9.1 (SAS Institute, Cary, NC, USA). Statistical significance was set at *P *< 0.05.

## Results

Of the mothers, 1.88% had less than a middle school education, 32.9% had finished high school, and 65.2% had more than a high school education. Of the fathers, the respective percentages were 1.32%, 22.5%, and 76.2%. Parental education was classified as low (<12 years or less than a high school education) or high (>12 years or more than a high school education) (data not shown). Table 1 shows the demographic and anthropometric characteristics of study participants according to the factors comprising SES. Children's WBCs, SBP (systolic blood pressure), and DBP (diastolic blood pressure) differed significantly according to paternal education and household income, but no significant differences were observed in BMI, height, weight, hb levels, iron levels, or the prevalence of anemia and iron deficiency. Children of less educated mothers had higher WBCs (*P *= 0.0005), ALT (*P *= 0.0320), SBP (*P *< 0.0001), and DBP (*P *= 0.0005). They also had lower indices of iron status, serum iron, TIBC, TS, and hb compared with children of more educated mothers, although the differences were not statistically significant. A rates of of anemia and iron deficiency were found among children of less educated mothers (10.8 *vs. *6.2%, *P *= 0.0324 and 4.48 *vs. *1.78%, *P *= 0.0577, respectively) than among children of more educated mothers. Overall, we observed a lower prevalence of iron deficiency than of anemia. Only two children had both low serum hb levels and low iron levels. Low hb and/or low iron levels were found in 10.5% of this population.

**Table 1:** Characteristics of the subjects according to maternal education, paternal education, and household income

Variable	Maternal education					Paternal education					Household income				
	
	Low (*n *= 268)		High (*n *= 449)			Low (*n *= 191)		High (*n *= 526)			Low (*n *= 207)		High (*n *= 510)		
	
	Mean	SD	Mean	SD	*P*	Mean	SD	Mean	SD	*P*	Mean	SD	Mean	SD	*P*
Sex (M/F), *n*	135/133		227/222		0.9620^**a**^	94/97		268/258			95/112		267/243		
Height	140.0	6.66	140.1	5.93	0.0516^**b**^	139.9	6.74	140.1	6.01	0.0880	140.0	6.83	140.0	5.95	0.9524
Weight	36.7	8.23	36.1	7.18	0.9935	36.7	8.01	36.2	7.43	0.9508	35.9	7.77	36.5	7.52	0.5912
BMI (kg/m^2^)	18.6	3.14	18.3	2.80	0.3404	18.6	3.06	18.3	2.89	0.3861	18.2	2.81	18.5	2.98	0.4619
WBC	6.8	1.87	6.3	1.50	0.0005	6.8	1.92	6.4	1.54	0.0052	6.7	1.83	6.4	1.58	0.0352
RBC	4.64	0.61	4.72	0.47	0.2433	4.68	0.57	4.70	0.51	0.6494	4.65	0.59	4.71	0.50	0.1739
Glucose	86.5	7.3	85.1	7.0	0.1043	86.9	7.7	85.1	6.9	0.0366	85.6	7.9	85.6	6.8	0.8853
ALT	16.5	17.3	14.7	6.9	0.0320	15.9	15.1	15.2	10.6	0.3626	14.5	11.3	15.7	12.2	0.3264
SBP	108.0	13.0	100.3	12.3	<.0001	109.7	13.4	100.8	12.2	<.0001	107.0	13.0	101.6	12.8	<.0001
DBP	70.4	8.8	67.7	9.2	0.0005	71.3	8.8	67.7	9.1	<.0001	69.9	9.1	68.2	9.1	0.0116
Iron (μg/dl)	99.3	36.3	101.6	34.8	0.3363	98.3	36.2	101.6	35.1	0.2217	98.0	36.6	101.8	34.8	0.1498
Total iron-binding capacity (μg/dl)	340.2	61.7	345.8	47.9	0.0589	338.2	65.3	345.7	48.4	0.0321	341.5	59.0	344.6	51.1	0.4808
TS (%)	29.5	10.4	29.6	10.1	0.9351	29.4	10.1	29.7	10.3	0.7729	29.0	10.6	29.8	10.0	0.2845
Ferritin (μg/l)	40.1	20.2	38.7	20.0	0.5313	38.8	20.7	39.4	19.8	0.5076	38.2	20.5	39.7	19.9	0.4127
Serum transferrin receptor (mg/l)	2.13	0.72	2.05	0.54	0.0587	2.13	0.72	2.06	0.57	0.1051	2.11	0.66	2.07	0.59	0.2546
Hemoglobin (g/dl)	13.0	1.64	13.3	1.26	0.0660	13.1	1.53	13.2	1.38	0.5260	13.1	1.58	13.2	1.35	0.0785
**Anemia**^**c **^**(*n*, %)**	29 (10.8)		28 (6.2)		0.0324^**d**^	20 (10.5)		37 (7.0)		0.1589	20 (9.7)		37 (7.3)		0.2883
**Iron deficiency**^**e **^**(*n*, %)**	12 (4.48)		8 (1.78)		0.0577	9 (4.71)		11 (2.09)		0.0724	9 (4.35)		11 (2.16)		0.1318
**Iron-deficiency anemia**^**f **^**(*n*, %)**	1 (0.37)		1 (0.22)			0		2 (0.38)				0	2 (0.39)		
**Anemia & iron deficiency**	40 (14.9)		35 (7.8)		0.0035	29 (15.2)		46 (8.8)		0.0183	29 (14.0)		46 (	9.0)	0.0588

*BMI *body mass index

^*a*^*P *value by *chi-square *test

^b ^*P*-value by generalized linear model adjusted by age and sex

^c^Anemia: hemoglobin <11.5 g/dl^(1)^

^*d*^*P *value by Fisher's exact test

^e^Iron deficiency: iron < 40 μg/dl^(22)^

^f^Iron-deficiency anemia: hemoglobin <11.5 g/dl and iron <40 μg/dl

Subjects were measured for SBP and DBP by maternal educational level (low = 267, high = 448), paternal educational level (low = 190, high = 525), and household income level (low = 205, high = 510)

As shown in Table 2, children of less educated mothers consumed less energy (1649.0 *vs. *1746.7 kcal, *P *= 0.0003), fat (45.1 *vs. *50.3 g, *P *< 0.0001), carbohydrates (245.5 *vs. *253.6 g, *P *= 0.0379), animal protein (33.5 *vs. *37.9 g, *P *< 0.0001), vitamin B1 (1.2 *vs. *1.3 mg, *P *= 0.0214), niacin (15.2 *vs. *16.2 mg, *P *= 0.0120), zinc (8.2 *vs. *8.8 mg, *P *= 0.0336), phosphorus (958.8 *vs. *997.6 mg, *P *= 0.0425), animal iron (2.9 *vs. *3.2 mg, *P *= 0.0012), and fat derived from energy (24.5 *vs *25.6%, *P *= 0.0067) than did children of more educated mothers. The opposite pattern was observed with regard to the intake of fiber, vitamin C, and folate. The mean iron intake w greater than the daily estimated average requirement set by the Korean iron EAR criteria. However, children of less educated mothers were less likely to surpass the iron EAR levels tha were children of more educated mothers (20.9 *vs. *15.4%, *p *= 0.0591). With respect to food-gro consumption, children of more educated mothers were less likely to consume vegetables (258. *vs. *223.5 g, *P *< 0.0001). However, more meat, poultry, and their derived products (77.7 *vs. *99.8 g, *P *< 0.0001) as well as more fish and shell fish (43.6 *vs *49.5 g, *P *= 0.0362) were consumed by children of more educated mothers.

**Table 2:** Nutrient and food-group intake of subjects by maternal education level

Nutrients	Low (*n *= 268)		High (*n *= 449)		***P*-value **^**a**^
		
	Mean	SD	Mean	SD	
Energy intake (kcal)	1649.0	344.9	1746.7	370.5	0.0003
Fat (g)	45.1	15.0	50.3	17.2	<.0001
Carbohydrate (g)	245.5	55.5	253.6	51.9	0.0379
Protein (g)	66.6	15.7	71.3	18.9	0.0004
Plant protein (g)	33.1	8.0	33.4	8.5	0.5566
Animal protein (g)	33.5	11.7	37.9	15.7	<.0001
Vitamin A (μg RE)	736.5	352.1	712.1	308.0	0.2286
Vitamin B_1 _(mg)	1.2	0.4	1.3	0.4	0.0214
Vitamin B_2 _(mg)	1.1	0.4	1.1	0.4	0.4561
Niacin (mg)	15.2	4.9	16.2	5.4	0.0120
Vitamin B_6 _(mg)	1.9	0.6	1.9	0.6	0.4019
Vitamin C (mg)	85.0	43.4	75.3	35.8	0.0038
Vitamin E (mg)	12.7	4.6	12.9	4.8	0.6882
Folate (μg)	234.1	85.7	216.3	75.5	0.0068
Zinc (mg)	8.2	3.0	8.8	3.5	0.0336
Calcium (mg)	512.5	187.1	498.0	198.5	0.4101
Phosphorus (mg)	958.8	240.1	997.6	268.0	0.0425
Sodium (mg)	4051.9	1259.8	3914.6	1147.8	0.1776
Potassium (mg)	2504.3	757.1	2456.1	697.5	0.2910
Iron (mg)	11.8	3.5	12.1	3.4	0.2981
Plant iron (mg)	9.0	3.0	8.9	2.9	0.7432
Animal iron (mg)	2.9	1.1	3.2	1.5	0.0012
Fiber (g)	18.1	7.9	16.6	5.0	0.0038
Cholestrol (mg)	292.2	140.5	305.9	141.8	0.1112
**% Energy**					
Carbohydrate (%)	59.6	5.9	58.4	6.0	0.0101
Protein (%)	16.2	2.1	16.3	2.2	0.5912
Fat (%)	24.5	5.4	25.6	5.5	0.0067
**Low iron intake (*n*, %)**^b^	56	(20.9)	69	(15.4)	0.0591
Food group (g)					
Cereal & grain products	271.1	73.0	285.8	75.4	0.0051
Potatoes & starch	37.8	36.9	39.4	44.7	0.6973
Sugars & sweet	5.8	5.1	6.5	7.0	0.1781
Legumes & their products	35.6	37.7	32.6	32.5	0.4730
Seeds & nuts	3.7	8.6	3.0	7.8	0.4275
Vegetables	258.0	114.6	223.5	96.0	<.0001
Fungi and mushroom	3.3	12.4	2.9	9.9	0.6945
Fruit	128.7	147.7	134.0	138.9	0.9898
Meat, poultry, & their products	77.7	50.4	99.8	73.6	<.0001
Eggs	30.5	25.8	30.2	24.2	0.7095
Fish & shell fish	43.6	31.9	49.5	40.0	0.0362
Seaweed	3.2	5.2	2.9	5.2	0.6127
Milk & milk products	133.3	119.9	121.2	121.7	0.2809
Beverages	46.4	41.2	44.9	37.9	0.8627
Oil (plant)	8.7	4.2	8.8	4.5	0.4033
Fat (animal)	0.2	1.0	0.2	0.7	0.4738

*RE *retinol equivalent

^a ^*P*-value by generalized linear model adjusted by age and sex

^b ^Iron EAR criteria: 9 mg/day

Table 3 shows the distributions of food-consumption behaviors according to the level of maternal education. We found significant differences between the groups in the frequencies of drinking carbonated beverages (66.2 *vs. *55.7%, *P *= 0.0061) and eating instant noodles (74.6 *vs. *67.4%, *P *= 0.0416). No significant differences were observed with regard to the consumption of fast food.

**Table 3:** Food-consumption behavior by maternal education level

Variables (occasionally/weekly)	Low (*n *= 268)		High (*n *= 449)		***P*-value **^**a**^
			
	*n*	%	*n*	%	
Eat fast food					0.5506
No	129	48.5	206	46.2	
Yes	137	51.5	240	53.8	
Drink carbonated beverages					0.0061
No	90	33.8	197	44.3	
Yes	176	66.2	248	55.7	
Eat instant noodles					0.0416
No	68	25.4	145	32.6	
Yes	200	74.6	300	67.4	

^a ^*P*-value by *chi*-square test

Table 4 shows the odds ratios (ORs) and 95% confidence intervals (CIs) for the occurrence of anemia and iron deficiency in children according to level of maternal education. According to an unadjusted model, overweight (OR: 1.73; 95% CI: 1.02, 2.96) and dietary vitamin C (OR: 1.01; 95% CI: 1.00, 1.01) were significant predictors of anemia and iron deficiency. Children of more educated mothers had a lower risk of anemia or iron deficiency (OR: 0.48; 95% CI: 0.30, 0.78). In the adjusted analysis, maternal education was the only statistically significant predictor of anemia or iron deficiency (OR: 0.52; 95% CI: 0.32, 0.85).

**Table 4:** Logistic regression analyses predicting odds ratios of anemia and iron deficiency

Variables	Logistic regression predicting	**anemia and iron deficiency**^**a**^
	
	**Unadjusted**^**b **^**OR**^**c **^**(95% CI**^**d**^**)**	**Adjusted**^**e **^**OR (95% CI)**
Maternal education^f^	0.48 (0.30-0.78)**	0.52 (0.32-0.85)**
Overweight ^g^	1.73 (1.02-2.96)*	
Dietary fiber	1.02 (0.99-1.06)	
Dietary animal protein	1.00 (0.98-1.02)	
Dietary animal iron	1.05 (0.89-1.23)	
Dietary vitamin C	1.01 (1.00-1.01)*	
Dietary folate	1.00 (1.00-1.01)	
Dietary zinc	0.99 (0.92-1.07)	
Dietary fat	1.00 (0.99-1.02)	

^a ^Logistic regression was used to predict yes or no answers to a question about whether a subject had anemia or iron deficiency

^b ^Unadjusted analysis refers to a simple logistic regression with the dependent variable

^c ^*OR *odds ratio

^d ^*CI *confidence interval

^e ^Adjusted analysis refers to the covariates that were best able to predict poor health, anemia, and iron deficiency using stepwise regression techniques in a multivariable model

^f ^Maternal education (1, low; 2, high)

^g ^Overweight (1, BMI < 85th percentile; 2, BMI ≥ 85th percentile)

*P < 0.05, ** *P *< 0.01

## Discussion

We investigated the relationship between the factors comprising SES (particularly maternal education) and a school-aged children's diet, their risk for anemia, and their risk for iron deficiency in Korea. Overall, Korea's economy is classified between developing and developed. The rates of anemia and iron deficiency were 14.9% in the low-maternal-education group and 7.8% in the high-maternal-education group in Korea. The relationship between maternal education and anemia/iron deficiency has been evaluated in industrialized countries. Anemia and iron deficiency were found to be problems in industrialized nations [1], and socioeconomic inequalities may affect the prevalence of disease by influencing dietary choices [25]. Recently, Korea has experienced remarkable economic growth, but social inequalities remain [26]. Different dietary patterns may result from differences in the factors comprising SES [27], which may affect the prevalence of chronic diseases.

To our knowledge, this study is the first to investigate the association of maternal education with dietary intake and indices of anemia/iron status in Korean school-aged children. Many studies have separately explored the association of the factors comprising SES with anemia or nutrient intake. In the present study, only maternal education was found to have a significant relationship with hb levels and the prevalence of anemia. Iron deficiency was also related to maternal education, although the differences were only marginally significant. In this study, paternal education and household income were not significantly associated with anemia, iron deficiency, or hb levels. Other studies have suggested that maternal education was more important than paternal education, health-service availability, and other factors related to SES [28,29]. In this study, children with more educated mothers had lower WBCs, glucose and ALT levels, and lower blood pressure than did children with less educated mothers.

In terms of dietary factors, anemia is induced by lower levels of the consumption of dietary iron derived from foods such as meat and less intake of the nutrients involved in iron metabolism such as vitamins A and C. The presence of iron-absorption inhibitors (such as phytates in bran, calcium in dairy products, polyphenols in certain vegetables, and tannins in tea) [30] also play a role in anemia. In the present study, subjects in the low-maternal-education group reported a greater intake of fiber, which may have decreased the bioavailability of iron. The consumption of energy, fats, carbohydrates, animal protein, vitamin B_1_, niacin, zinc, phosphorus, and animal iron was significantly lower in the low-maternal-education group than in the high-material-education group. Additionally, children in the low-maternal-education group consumed more energy from carbohydrates and less energy from fat than did children in the high-maternal-education group.

According to the Korean National Health and Nutrition Examination Survey-III (KNHANES III), the major food sources of iron were almost exclusively derived from Korean plant sources. Rice was the primary source of iron; this is followed by radish leaves and kimchi (which contain small amounts of protein and heme iron) [31]. Similarly, our study showed that children of less educated mothers consumed less meat, poultry (and their derivatives), fish, and shell fish but consumed more vegetables. The major cause of anemia may be a diet low in meat, fish, or poultry [30]. Heme iron from hemoglobin and myoglobin found in meat, fish, and poultry are effectively absorbed by receptors in the gut, whereas the bioavailability of non-heme iron from plants is low. Differences in food-group consumption were observed in this study in terms of the smaller quantities of food derived from animal sources that were consumed by children in the low-maternal-education group. These results explain why children of less educated mothers showed a higher prevalence of anemia.

Our study shows that children of mothers with more education were less likely to have unhealthy eating patterns (e.g., drinking carbonated beverages and eating instant noodles) than were children of mothers with less education. Several studies have reported that a higher intake of carbonated drinks was associated with a higher intake of energy and carbohydrates as well as a lower intake of protein, vitamins and minerals [32]. Thus, a relationship between the educational level of mothers and the quality of the diet of their children has been observed. Children with less educated mothers consumed less of most vitamins and minerals and were less likely to have consumed a range of "health foods" [33]. It is possible that the food-consumption behavior of children may lead to both iron deficiency and obesity [34]. Specific habits, such as snacking and eating junk food, may also contribute to anemia. People with higher levels of education have better eating habits [14] and may therefore be healthier.

In this study, BMI was not affected by maternal education, but being overweight was a significant risk factor for anemia and iron deficiency in the unadjusted logistic regression model. Although some studies have reported an association between maternal education and the likelihood of a child being overweight [28,35], no studies have demonstrated an association between maternal education and hb levels or between the prevalence of both anemia and iron deficiency and the likelihood of being overweight.

We found the maternal education plays important role in the nutritional status of children among SES factors in this study. Children with more highly educated mothers were less likely to suffer from anemia and iron deficiency than were children with less educated mothers. A higher level of maternal education leads to increased knowledge about health and nutrition, which, in turn, leads to an increase in the quality of the diets consumed by children [15]. This study found a significant relationship between maternal education and dietary factors and between maternal education and the likelihood of children suffering from anemia and iron deficiency. Indeed, many studies have shown that maternal education affects children's diets and that this directly influences children's health [14,15,28,35]. We also found that maternal education independently affected the likelihood of children developing anemia and/or iron deficiency. This study has three main strengths. First, dietary intake was surveyed using a 3-day food diary. Second, this study is the first to investigate the complex relationship between maternal education and children's diets and the biochemical indices of their iron status. Third, we observed a significant relationship between maternal education and childhood anemia/iron deficiency even after adjusting for potential confounding factors.

Our study has the following limitations. Data on parental education were obtained from parental self reports, which may have resulted in an overestimation bias. Additionally, because we could not inquire about mothers' knowledge of nutrition, we could not analyze the direct association between maternal education and nutrition-related knowledge. However, maternal education level was associated with children's dietary intake, food-consumption behaviors, and likelihood of suffering from anemia/iron deficiency. Maternal education was the strongest predictor of all of the factors comprising SES that we evaluated. We measured serum ferritin, sTfR, iron, TS, hb, and dietary iron intake to ascertain iron status. Because the majority of subjects was in good health and well nourished, iron deficiency was not easily detected. However, subjects with low hb levels were more prevalent than were subjects with low iron indices. Finally, our study was cross-sectional in design; additional studies using a prospective design and measurements before and after interventions are required to fully determine the causal relationships among maternal education, children's diet, and disease.

## Conclusions

Of the SES factors that we evaluated, only maternal education was related to children's hb levels and the prevalence of anemia and iron deficiency. More maternal education was positively associated with children's consumption of foods that affect the bioavailability of iron and reduce the risk of anemia and iron deficiency. To increase the absorption of iron and prevent anemia and iron deficiency, mothers should feed children foods that enhance iron absorption. The results of this study should be helpful in future investigations of anemia, nutrition, and SES.

## Competing interests

The authors declare that they have no competing interests.

## Authors' contributions

H-JC and H-BJ performed data analysis and produced the results and H-JC drafted the manuscript. H-JL and J-YP have been involved in critically revising the manuscript for important intellectual content. H-JL, J-HK, K-HP and JS contributed to the conception of the study and participated in the interpretation of the results and revision of the manuscript. All authors read and approved the final manuscript.

## Pre-publication history

The pre-publication history for this paper can be accessed here:

http://www.biomedcentral.com/1471-2458/11/870/prepub
